# The Genetics of Diabetes: What We Can Learn from *Drosophila*

**DOI:** 10.3390/ijms222011295

**Published:** 2021-10-19

**Authors:** Francesco Liguori, Elisa Mascolo, Fiammetta Vernì

**Affiliations:** 1 Preclinical Neuroscience, IRCCS Santa Lucia Foundation, 00143 Rome, Italy; f.liguori@hsantalucia.it; 2 Department of Biology and Biotechnology “Charles Darwin”, Sapienza University, 00185 Rome, Italy; elisa.mascolo@uniroma1.it

**Keywords:** diabetes, *Drosophila*, glucose homeostasis, insulin signaling

## Abstract

Diabetes mellitus is a heterogeneous disease characterized by hyperglycemia due to impaired insulin secretion and/or action. All diabetes types have a strong genetic component. The most frequent forms, type 1 diabetes (T1D), type 2 diabetes (T2D) and gestational diabetes mellitus (GDM), are multifactorial syndromes associated with several genes’ effects together with environmental factors. Conversely, rare forms, neonatal diabetes mellitus (NDM) and maturity onset diabetes of the young (MODY), are caused by mutations in single genes. Large scale genome screenings led to the identification of hundreds of putative causative genes for multigenic diabetes, but all the loci identified so far explain only a small proportion of heritability. Nevertheless, several recent studies allowed not only the identification of some genes as causative, but also as putative targets of new drugs. Although monogenic forms of diabetes are the most suited to perform a precision approach and allow an accurate diagnosis, at least 80% of all monogenic cases remain still undiagnosed. The knowledge acquired so far addresses the future work towards a study more focused on the identification of diabetes causal variants; this aim will be reached only by combining expertise from different areas. In this perspective, model organism research is crucial. This review traces an overview of the genetics of diabetes and mainly focuses on *Drosophila* as a model system, describing how flies can contribute to diabetes knowledge advancement.

## 1. Glucose Homeostasis Maintenance

Diabetes is a chronic metabolic disease affecting more than 450 million people worldwide. It can be considered as a group of diseases characterized by increased glucose levels in the blood leading, over the time, to serious damage in different organs, such as heart, blood vessels, eyes, kidneys and nerves. Although different types of diabetes exist, all forms are attributable to two main causes: impaired insulin secretion and/or a reduced response of cells to the insulin action [1].

**Figure 1:**
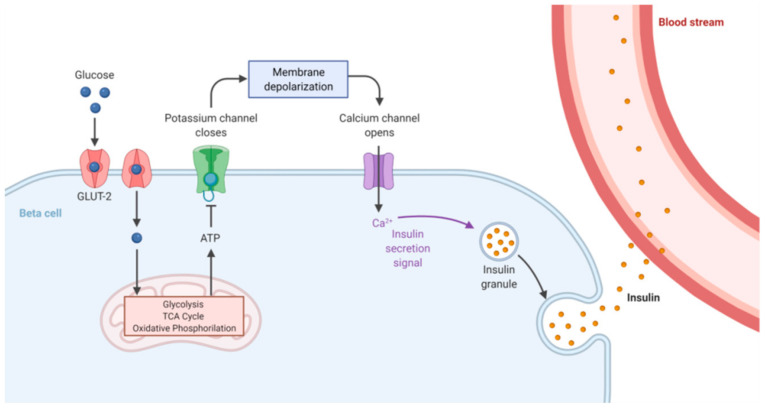
Insulin secretion mechanism. Glucose is transported into pancreatic beta cells through facilitated diffusion by GLUT2 glucose transporters. Intracellular glucose is metabolized to ATP through glycolysis and oxidative phosphorylation. A high ATP/ADP ratio induces closure of K^+^ channels, leading to cell membrane depolarization. This event promotes the opening of Ca^2+^ channels, facilitates extracellular Ca^2+^ influx into the beta cell, and in turn triggers insulin exocytosis. Adapted from “Insulin Production Pathway”, by BioRender.com (2021). Retrieved from https://app.biorender.com/biorender-templates, accessed on 15 September 2021.

The levels of glucose in the blood need to be tightly maintained into a well-defined range in order to guarantee the availability of this sugar as source of energy and contextually prevent organ damage due to its excessive concentration. Glucose homeostasis is therefore based on the combined and opposite action of two hormones, insulin and glucagon, which, in mammals, are secreted from beta and alpha pancreatic cells, respectively [2]. Insulin decreases glucose concentration in the bloodstream by promoting its uptake and utilization through the glycolytic pathway, whereas glucagon promotes the synthesis of free glucose from glycogen (the glucose storage form). Complex mechanisms, not yet completely understood, finely regulate this balance. Insulin secretion depends on the open or closed status of the ATP-dependent potassium channels (K^+^ ATP channels); when blood glucose levels are high, pancreatic beta cells promote glycolysis, a process leading to increased intracellular ATP concentration, and consequently K^+^ ATP channel inhibition and cellular membrane depolarization. This event opens the voltage-dependent calcium channels and the entering of Ca^2+^ ions enables insulin release into the bloodstream (Figure 1).

**Figure 2:**
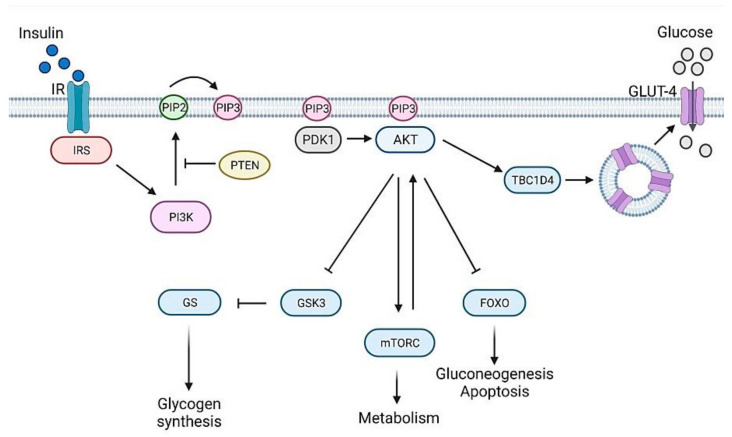
The mammalian insulin signaling pathway, a simplified schematic representation. Insulin receptor (IR) through insulin receptor substrate (IRS) creates binding sites that recruit the lipid kinase phosphoinositide 3-kinase (PI3K) to the plasma membrane. PI3K phosphorylates phosphatidylinositol 4,5-bisphosphate (PIP2) to produce phosphatidylinositol 3,4,5-trisphosphate (PIP3), which can be dephosphorylated back to PP2 by phosphatase and tensin homologue (PTEN), a lipid phosphatase. PIP3 recruits phosphoinositide-dependent kinase 1 (PDK1) and serine/threonine protein kinase AKT to the plasma membrane, where AKT is fully activated by PDK1 and by mechanistic target of rapamycin complex (mTORC) protein kinases. AKT controls cellular metabolism through key downstream substrates. In particular, AKT inhibits the activity of GSK3, thus stimulating the glycogen synthesis by glycogen synthase (GS). In addition, it promotes the translocation of GLUT4 to the plasma membrane, through TBC1 domain family member 4 (TBC1D4), thus favoring glucose uptake; AKT prevents gluconeogenesis and apoptosis by controlling the activity of forkhead box O (FOXO) transcription factors; AKT controls cell growth and metabolism by regulating mTORC activity. Figure 2 was created with BioRender.com (https://app.biorender.com, accessed on 15 September 2021).

Once produced, insulin is delivered to target tissues (liver, adipose cells, muscles, brain) where it binds insulin receptor (IR) and triggers a cascade of phosphorylation events, named insulin/insulin-like growth factor signaling (IIS) (Figure 2), ultimately leading to glucose uptake and storage in the form of glycogen, thus decreasing glucose blood levels. The name IIS clearly indicates that the same pathway may be activated not only by insulin but also by insulin-like growth factors (IGFs) through binding to their specific receptors [3]. After its activation, insulin receptor (IR) phosphorylates several substrates including insulin receptor substrate (IRS) protein which provides specific docking sites for phosphatidylinositol 3-kinase (PI3K) activation. This enzyme generates phosphatidylinositol (3,4,5)-triphosphate (PIP3), which in turn recruits phosphoinositide dependent protein kinase 1 (PDK1) and AKT to the plasma membrane, where PDK1 activates AKT. PI3K activity is counteracted by the activity of PTEN (phosphatase and tensin homolog) [4]. Full AKT activation also requires the activity of the mammalian target of rapamycin complex (mTORC2) [5,6]. Once activated, AKT phosphorylates several downstream targets such as GSK-3 and TBC1D4, responsible, respectively, for glycogen synthesis and glucose uptake through GLUT4 glucose transporter translocation. Other AKT targets are the rapamycin complex (mTORC1), involved in cellular growth, and FOXO proteins, whose expression impacts on gluconeogenesis and apoptosis [7]. IIS is finely regulated by negative feedback signals. The mTORC1 and S6-kinase (S6K) complex—downstream components of the pathway [8]—phosphorylates mTORC2, attenuates its activity and hence reduces AKT action [9,10]. More recently, Kearney and collaborators showed that AKT-mediated post-translational modifications of the IRS represent another feedback signal that controls PIP3 abundance [11].

## 2. Genetics of Diabetes

**Figure 3:**
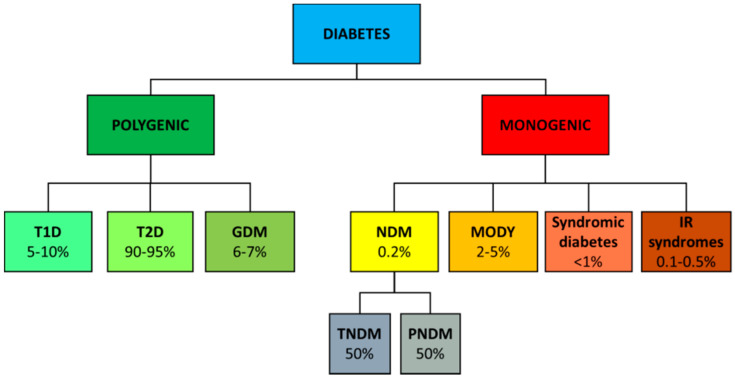
Classification of diabetes. Different diabetes and their rough frequencies.

A classification of the different diabetes forms is shown in Figure 3. Most common forms of diabetes such as type 1 (T1D), type 2 (T2D) and gestational diabetes (GDM) are complex diseases determined by many genes influenced by environmental factors. Rare forms of diabetes, including neonatal (NDM) and maturity onset diabetes of the young (MODY) are, instead, due to single gene mutations. 

### 2.1. Type 1 Diabetes

Type 1 diabetes (T1D), previously known as juvenile diabetes, is characterized by impaired insulin secretion; it represents 5–10% of all diabetes cases and needs to be treated with insulin. T1D has been defined as an autoimmune disease because it is characterized by T-cell mediated autoimmune pancreatic beta cell destruction [12]. 

T1D is a multifactorial disease with a strong genetic component, since concordance rate is 30–70% in identical twins and the risk for children with a diabetic parent ranges from 1 to 9% [13,14]. 

Rapid technological advancement in the field of human genomics led to the identification of several T1D loci [15]. In particular, genome-wide association studies (GWAS) identified more than 60 susceptibility regions associated to T1D marked by single-nucleotide polymorphisms (SNPs). SNPs located within the human leukocyte antigen (HLA) region on chromosome 6 were found to confer the major heritable risk (~50%) for T1D [16]. In addition to HLA genes, more than 50 other genes have been associated with T1D. Most of these genes are related to immune functions and their identification allowed many cellular pathways to be recognized as pivotal for diabetes development, such as insulin gene expression in the thymus, regulation of T-cell activation and viral responses [14,17,18]. To date, however, the functional roles of most T1D-associated genetic variants still need to be determined. In addition, the 90% of genetic variants linked to T1D lie outside the coding regions [19], falling within regulatory loci, such as enhancer regions [20,21,22]. Deeper understanding of these HLA and non-HLA genetic associations could lead to the identification of potential therapeutic targets or subgroups of patients that may benefit from a specific immune intervention.

### 2.2. Type 2 Diabetes

T2D currently accounts for 90–95% of all diabetic patients. It is characterized by impaired insulin action and often accompanied, over time, by reduced insulin secretion [1].

Initially, in T2D patients, insulin is regularly produced but it is not able to stimulate the signaling, thus establishing a condition known as insulin resistance. The compromised response of target tissues to the hormone leads the pancreas to increase the rate of insulin secretion; however, over time, pancreas functionality decreases and insulin production is strongly reduced. Moreover, most, but not all, patients with type 2 diabetes are overweight or obese (excess weight itself may determine insulin resistance). T2D usually does not require insulin treatment but, as T1D, it can lead to a wide spectrum of micro- and macrovascular complications [1].

Studies aimed at exploring the genetic architecture of T2D revealed that it results from a strong hereditary component influenced by environmental exposures experienced throughout the lifespan; in addition, epigenetic factors might play an important role [23]. T2D hereditability ranges from 20% to 80% as estimated from population, family and twin-based studies [24,25]. Different approaches have been employed to identify T2D risk genes throughout the years. Candidate gene approach and linkage-based studies were the first exploited strategies, but they identified only a small number of susceptibility genes, such as *HNF4A* [26] and *TCF7L2* [27], which have been also replicated later. Subsequently, the advent of genome-wide association studies—focused on SNP recognition—followed by meta-analyses, led to the identification of hundreds of genes associated to T2D (*TCF7L2*, *SLC30A8*, *HHEX*, *ADAMTS9*, *CDC123*/*CAMK1D*, *CDKAL1*, *CDKN2A/B*, *IGF2BP2*, *JAZF1*, *NOTCH2*, *RBMS1*, *THADA*, *TSPAN8/LGR5*, *PPARG*, etc.) [28,29,30,31,32,33,34]. Despite this abundance, none of the numerous genetic variants were sufficiently penetrant to be considered as primary T2D cause.

The comprehension of how SNPs associated to T2D physiologically impact on its pathogenesis is still preliminary, and, on top of that, the majority of T2D-associated SNPs resulted in intergenic or intronic regions. However, in few cases, including *SLC30A8* and *TCF7L2*, the causative link between T2D and SNPs has been elucidated [35,36,37]. Surprisingly, different studies showed that T2D-associated SNPs impact more on insulin secretion rather than insulin resistance [38,39]. However, despite the enormous number of identified T2D-associated SNPs, it has been estimated they can explain less than 15% of T2D hereditability [40], thus conferring a low predictive power. 

Hence, to explain the “missing heritability”, genes located near the most associated SNPs have been re-sequenced with the aim to identify rare coding mutations putatively implied in T2D physiopathology and to establish causality relationships. However, these studies provided only limited evidence of the involvement of lower-frequency variants in T2D predisposition; they explain, at most, 25% of the heritability of the strongest common single-variant signals [41,42]. Nevertheless, this approach led to the discovery rare risk alleles in some genes, including *SLC30A8*, *MTNR1B* and *PPARG*, which can contribute to diabetes onset [43,44,45].

From all these studies, it emerges that the implementation of strategies aimed at investigating the functional roles of T2D-associated genes will be helpful to reveal new involved pathways and to potentially provide new therapeutic targets. In addition, some studies revealed that different variants of the same gene can differently respond to antidiabetic drugs [46,47]. However, although inter-individual differences are significantly associated with genetic makeup, thus playing a determining role in respect to disease susceptibility, it is also necessary to evaluate the role and the impact of the environmental factors, in order to make the application of precision medicine strategies feasible.

### 2.3. Gestational Diabetes

One of the most common complications of pregnancy is gestational diabetes mellitus (GDM), which affects 5% of pregnant women [1]. During pregnancy, insulin resistance can be considered almost as a physiological process aimed at ensuring the correct glucose intake to the growing fetus. For this reason, the pancreas of a pregnant woman is stimulated to produce a higher amount of insulin. In some women, pancreatic beta cells are unable to sustain this increased production of insulin, and so blood sugar levels are not kept within the normal range, thus causing the hyperglycemia that characterizes GDM. Differently from T2D, GDM often resolves after childbirth. However, sometimes GDM can represent a strong risk factor for T2D after pregnancy [48]. Untreated GDM can lead to adverse outcomes for both mother and child during pregnancy and childbirth [49].

Several approaches aimed at identifying the genetic factors predisposing to GDM, such as candidate gene approach and GWAS, led to the identification of numerous genes also involved in T2D pathogenesis, thus attesting that both diseases share a common pathophysiology [50]. A very strong association was found for *TCF7L2*, *MTNR1B*, *CDKAL1*, *IRS1* and *KCNQ1* genes which appeared the most diffused, while some others were confined to specific ethnic groups [51,52]. In particular, maternal *GCK* and *TCF7L2* variants carry an increased risk of adverse pregnancy outcome in women without overt diabetes [53]. However, to date, all the GDM-associated variants showed only a modest effect. 

Furthermore, the application of the aforementioned approaches is limited by the scarce availability of samples, due the low frequency of the disease. In addition, it also needs to be considered that GDM is a complex disease, hence influenced by epigenetic mechanisms and environmental components [50]. It is therefore worth also exploring other strategies to investigate the genetic predisposition to GDM. For example, studies concerning the impact of reduced levels of vitamin B6 on GDM gave interesting results indicating that vitamin B6 deficiency during pregnancy is a potential risk factor for gestational diabetes; moreover, they led to speculation that mutations in genes involved in B6 metabolism may increase individual GDM susceptibility [54,55,56]. Further studies in this direction, as well as other studies aimed at discovering new pathways, may provide new solutions to prevent or mitigate GDM and its complications for mother and child.

### 2.4. Monogenic Diabetes

Monogenic diabetes are rare forms caused by single gene mutations, that can be classified as neonatal diabetes mellitus (NDM), syndromic diabetes (not treated in this review) and maturity onset diabetes of the young (MODY). In addition, single mutations in genes belonging to the insulin pathway may lead to severe insulin resistance syndromes [57]. NDM emerges during the neonatal or infancy period, while MODY appears in adolescence or young adults before 25 years of age [58,59]. 

Whilst NDM is rare, affecting approximately 1 newborn out of 100,000 [60,61], MODY frequency ranges between ~1 and 4% of all cases of diabetes. NDM is further categorized into transient (TNDM) and permanent (PNDM), each one reporting a frequency of 50% [62]. TNDM often develops within the first few weeks of life and remits by a few months of age. However, relapse occurs in 50% of cases, typically in adolescence or adulthood. PNDM is characterized by persistent hyperglycemia within the first 12 months of life, generally requiring continuous insulin treatment. Clinical features of NDM also include intrauterine growth retardation, polyuria, severe dehydration and neurological disorders [57].

MODY patients exhibit mild or no diabetic symptoms; indeed, their higher glucose levels are often detected only during routine blood tests. In MODY, secretion of C-peptide is intact, beta cell antibodies are absent and body mass index is normal; therefore, usually, there is no need for insulin treatment. Only patients with some subtypes of MODY are prone to develop diabetes complications, thus influencing the decision to treat or not the disease [63]. 

#### 2.4.1. NDM

NDM is due to mutations in genes mainly affecting the functionality of pancreatic beta cells. The most frequent genetic causes of neonatal diabetes are abnormalities of the imprinted 6q24 locus (60–70% TNDM) and mutations of the *KCNJ11* and *ABCC8* genes [64,65,66]. Activating mutations in *KCNJ11* and *ABCC8* account for 12% and 13% of cases of TNDM, respectively, and for 31% and 10% of cases of PNDM [67]. *ABCC8* and *KCNJ11* genes encode for two components of the K^+^ ATP channels named Kir6.2 and SUR1, respectively. When these genes are mutated, K^+^ ATP channels remain permanently open, causing the block of insulin secretion [68].

Mutations of the insulin gene (*INS*) represent the third cause of NDM, by frequency, and have been found in both TNDM and PNDM. Most of the mutations are inherited in an autosomal dominant manner and affect the structure of pre-pro-insulin [69]. Mutant pro-insulin undergoes degradation in the endoplasmic reticulum (ER) and produces severe ER stress, which can lead to beta cell death or interfere with their growth and development [69]. Other *INS* mutations, transmitted in a recessive manner, alter, instead, the protein expression [70]. Rare mutations in other genes have also been associated to NDM [57]. In particular, homozygous mutations in glucokinase (*GCK*) gene have been mainly associated to PNDM [71,72]. Glucokinase acts as a sensor of glucose blood concentration, allowing the control of the secreted amount of insulin. In the homozygous state, these non-sense mutations cause NDM by completely impairing glucokinase-mediated glycolysis [72].

#### 2.4.2. MODY

MODY is predominantly inherited in an autosomal dominant manner. Fourteen subtypes of MODY are known, each one associated with mutations in a specific gene, as reported in Table 1. Remarkably, recent studies proposed to eliminate *BLK*, *PAX4* and *KLF11* genes [73] and to introduce *RFX6* [74]. Most MODY-associated genes, except *GCK*, encode for transcription factors involved in different manners in insulin secretion.

Mutations in *HNF1A*, *HNF4A*, and *GCK* genes account for about 95% of all MODY cases, although incidence rates vary among different populations. *HNF1A* and *HNF4A* encode hepatocyte nuclear factors involved in insulin expression, while *GCK* encodes for glucokinase enzyme which acts as a glucose sensor in pancreatic beta cells, playing a critical role in glucose homeostasis. Next-generation sequencing is the best way to diagnose MODY, because it allows the identification of more MODY subtypes with a single test. However, in less developed countries, because of the lack of resources and the high costs of sequencing technologies, up to 80% of MODY cases remain undetected or are misdiagnosed as T1D or T2D and incorrectly treated. 

Monogenic diabetes offers great opportunities to apply personalized medicine to patients; once recognizing the patients suspected to be affected by monogenic diabetes, the correct interpretation of sequenced variants is crucial to optimize the treatment and familial risk management. 

#### 2.4.3. Monogenic Forms of Insulin Resistance

Mutations in single genes can also cause severe forms of insulin resistance. These diseases can be loosely classified into two groups: diseases with primary disorders of insulin signaling, and diseases with primary defects in adipose tissue development or function (lipodystrophy) [75]. Several genes have been associated to these syndromes, including genes involved in IIS. Mutations of the *insulin receptor* gene (*IR*) can cause type A insulin resistance (TAIRS), Rabson–Mendenhall or Donohue syndromes. TAIRS is a rare disease (approximately 1 in 100,000) characterized by insulin resistance, hirsutism, *acanthosis nigricans*, or polycystic ovaries [76]. Rabson–Mendenhall syndrome and Donohue syndrome are characterized by severe insulin resistance and result in infant or pediatric death [77]. 

The second most common cause of single-gene insulin resistance is represented by mutations in the regulatory PI3KR1 subunit of the enzyme PI3K, which give rise to SHORT syndrome [78], characterized by short stature, inguinal hernia, ocular depression, teething delay and lipodystrophy. Interestingly, mutations affecting the genes encoding the protein kinase AKT2 or TBC1D4 have also been identified in families with severe insulin resistance, although these genes have not been associated yet with specific syndromes [79]. 

### 2.5. Other Diabetes

Finally, there is a variety of uncommon and diverse types of diabetes classified as “other diabetes” caused by chronic diseases, such as pancreatitis, cystic fibrosis or endocrinopathies, infections and drugs, extensively treated in [80].

## 3. *Drosophila* as a Diabetes Model 

Animal models have contributed enormously to the study of diabetes mellitus, offering to researchers the opportunity to examine in vivo genetic and environmental factors influencing the development of the disease and its complications. In addition, performing functional studies in animal models enables the generation of reproducible data and overcomes the limitations imposed by human research. Here we focused on *Drosophila* as a diabetes model (Table 2); other animal models have been extensively treated in other works (for a review see [81,82]).

In the last twenty years, *Drosophila melanogaster* has been widely used to dissect functional and structural components of the genome [83,84], to study the mechanisms underlying aging [85] and to characterize several human diseases, such as neurodegenerative [86,87,88], cardiovascular [89], renal [90] and metabolic ones, including diabetes [91]. The use of *Drosophila* in metabolism studies became possible after the discovery that flies and humans share most metabolic pathways [92]. The development of powerful genetic strategies combined to several resources—e.g., gene knock out and transgenic stocks—enables functional studies to be carried out in an effective, inexpensive and efficient way. In addition, flies offer the opportunity to realize large screening to individuate new genes and pathways involved in the physiopathology of the disease, ultimately representing potential drug targets. Finally, flies can be used as a cheap and time-saving screening platform in the preliminary steps of drug development. 

### 3.1. Glucose Homeostasis in Drosophila

Glucose homeostasis is maintained in a remarkably conserved manner in *Drosophila*. Flies possess insulin and glucagon counterparts which perform the same functions as the mammalian hormones. Eight genes encode *Drosophila* insulin-like peptides (DILPs), designated DILP1 to DILP8. Among these proteins, DILP2, DILP3 and DILP5 are involved in the regulation of hemolymph glucose levels and fat storage, and in the control of development, body size and longevity [93,94,95].

DILPs are secreted by a group of 14 specialized cells in the brain, named insulin producing cells (IPCs), representing the counterpart of the mammalian endocrine pancreas. The adipokinetic hormone (AKH) is, instead, the counterpart of glucagon and is produced by the *corpora cardiaca* (CC) cells in the neuroendocrine ring gland [96,97].

*Drosophila* owns an open circulatory system, the hemolymph, in which trehalose and glucose are the most abundant sugars; trehalose is made of two glucose molecules and is synthesized in the fat body, the fly organ corresponding to liver and adipose tissue. Trehalose levels are 100-fold higher than glucose [96], but its hemolymph concentration is regulated more flexibly than that of glucose; perhaps because, being a non-reducing sugar, its accumulation does not produce any toxic effect. In contrast, *Drosophila* glucose levels are tightly regulated so as in mammals [98,99].

In adult fly brains, DILPs are secreted by IPCs with mechanisms similar to those seen in mammals. Glucose-mediated activation of IPCs involves the closure of K^+^ ATP channels and the opening of Ca^2+^ channels, which triggers DILPs release [100,101].

**Figure 4:**
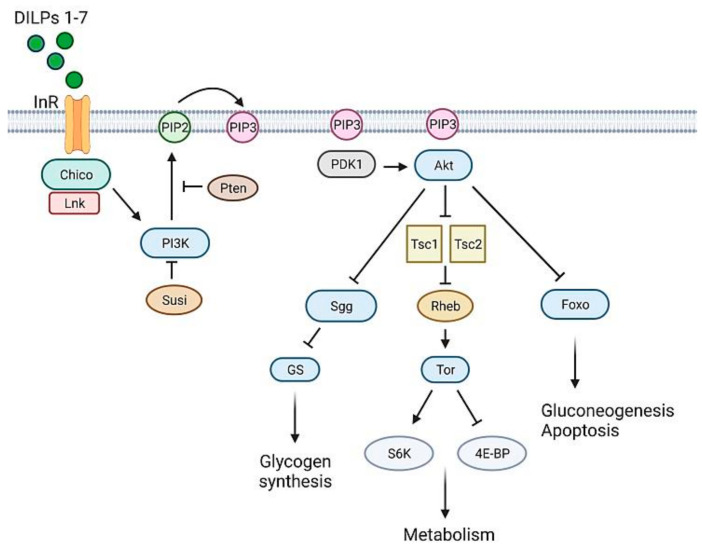
The *Drosophila* insulin signaling pathway, a simplified schematic representation. The *Drosophila* insulin-like peptides (DILP1-7) bind to insulin-like receptor (InR), which recruits the docking proteins Chico and Lnk. These proteins activate PI3K which converts PIP2 to PIP3 in the plasma membrane. The production of PIP3 is negatively regulated by PTEN and Susi. PIP3 recruits two kinases, PDK1 and Akt, to the plasma membrane enabling PDK1 to phosphorylate Akt. Akt mediates several signaling pathways essential for cell growth and survival, by acting on several downstream substrates, such as the transcription factor Foxo involved in metabolism and stress responses, the Tsc1/Tsc2 complex, an inhibitor of the Tor signaling pathway and essential regulator of growth and metabolism, and Sgg whose inactivation promotes the activity of glycogen synthase (GS). Figure 4 was created with BioRender.com (https://app.biorender.com, accessed on 15 September 2021).

The insulin signaling pathway is well conserved in flies [102] (Figure 4). Vertebrate and *Drosophila* insulin receptors are equivalent [103], as shown by the finding that chimeric fruit fly-vertebrate insulin receptors are activated with a similar mechanism [104]. However, different from mammals, InR is the only insulin receptor which mediates both energy metabolism and growth control [95].

The binding of DILPs 1-7 to insulin receptor (InR) leads to the recruitment of proteins such as Chico [105] and Lnk [106]. Chico is the IRS fly ortholog [105], Lnk (the fly ortholog of vertebrate SH2B adaptor proteins [107]) is an adaptor molecule that facilitates Chico and InR membrane localization [106]. As in mammals, Chico phosphorylation enables PI3K (a dimer composed by the catalytic Dp110 and the regulative Dp60 subunit) to create binding sites for PDK1, which is responsible for Akt activation [108]. The phosphorylating activity of PI3K is counteracted by the ortholog of PTEN [109]. In *Drosophila*, another negative regulator of PI3K is Susi, which binds to the Dp60 subunit [110]. Like in mammals, Akt acts on many different substrates [102], thus promoting glucose uptake, glycogen synthesis and protein synthesis. *Drosophila* does not have an ortholog of GLUT4, the glucose transporter which responds to insulin signaling. However, insulin-dependent glucose uptake seems to be a conserved process; indeed, transgenic flies expressing a human *GLUT4* in fat cells respond to mammalian insulin, promoting hGLUT4 trafficking and its translocation to the membrane [111]. 

### 3.2. T1D Fly Models

The first indication that *Drosophila* could be exploited as model for diabetes came from studies of Rulifson and collaborators [112] showing that ablation of IPCs increased the level of circulating sugars; moreover, this phenotype was rescued by DILP expression. This finding was also confirmed independently by other groups [113,114]. Notably, IPC-ablated animals were smaller and showed delayed development, indicating that also in *Drosophila*, insulin controls both growth and metabolism [112]. Later, Haselton and colleagues demonstrated that the OGTT test, employed routinely to diagnose human diabetes, can be applied to adult flies, thus reinforcing the evidence that flies can accurately model T1D [115]. 

Another T1D model was produced by depleting *dilp* genes using a deletion that simultaneously removed *dilps* 1-5. This deletion produced growth and development defects comparable to those observed in IPC-ablated flies. Moreover, flies displayed increased circulating sugars, small body size, decreased levels of triglycerides and reduced metabolic activities [116]. Studies exploiting different combinations of *dilp* mutants indicated that, in particular, *dilp 2*, *3* and *5* [113,114] are involved in the control of circulating sugar levels [93]. 

T1D is caused by progressive destruction of pancreatic beta cells. This cell damage causes insulin deficiency and deregulation of glucose metabolism. A consistent group of evidence indicates that ER stress is involved in beta cell destruction or malfunctioning, supported also by the finding that proteins involved in the ER stress response have been found altered in diabetic patients [117,118,119,120]. Starting from these premises, Katsube and co-workers tried to generate another *Drosophila* model to investigate the relationship between ER stress and IPC destruction. In this model, ER stress was induced in IPCs through the expression of a dominant negative form of the heat shock 70kDa protein cognate 3 (Hsc70-3), which is a *Drosophila* ortholog of the ER chaperone. This resulted in increased glucose levels and reduced DILP expression. Using this model, authors demonstrated that diabetes onset might be triggered by a cause–effect relationship between ER stress and induced destruction of IPCs mediated by apoptosis [121].

### 3.3. T2D Fly Models

T2D can be modeled in flies using different strategies. The first is the exposure to a high sugar diet (HSD) that produces hyperglycemia and hallmarks of insulin resistance [122]. HSD-fed larvae show enhanced expression of DILP2, DILP3, and DILP5, although they cannot activate insulin signaling [98,122]. Notably, HSD can also lead to obesity, characterized by increased lipid droplet size and triglyceride accumulation [122]. In addition, flies reared on an HSD-supplemented medium displayed delayed development, small body size and reduced lifespan. This last phenotype was recently attributed to a process of dehydration induced by HSD that promotes the accumulation of uric acid [123]. T2D is also induced by inactivating genes belonging to IIS, such as *InR*, *chico*, *PI3K* and *Akt* [105,124,125,126]. Given that mutations in IIS genes cause early lethality in homozygous condition, to overcome this problem they have been studied either in heteroallelic combinations or, alternatively, employing the RNAi-induced silencing in whole body or in specific tissues [125]. IIS mutants or RNAi flies display insulin resistance, elevated levels of glucose in the hemolymph, impaired lipid storage and reduced body size [105,124,125,126]. Similar to diabetic hyperinsulinemia, these flies exhibit increased DILP secretion [127]. Remarkably, although T2D is a polygenic disease in humans, this fly model recapitulates all insulin resistance hallmarks, thus allowing the study of the disease in a controlled genetic background.

Another T2D model has been produced through depletion of both Pyridoxal kinase (Pdxk) and Sugarlethal (Sgll), the fly ortholog of mammalian pyridoxine pyridoxamine oxidase (PNPO). The concerted action of these two enzymes synthesizes the active form of vitamin B6, the pyridoxal 5′-phosphate (PLP), which is a cofactor of more than 150 metabolic enzymes [128]. According to previous studies indicating that PLP levels are reduced in diabetic patients and diabetic animal models [129], larvae carrying homozygous *dPdxk^1^* mutations displayed insulin resistance due to reduced levels of Akt phosphorylation, were hyperglycemic, and died before reaching the pupal stage [130]. Conversely, flies in which *sgll* expression was reduced by RNA interference showed reduced adult body size, hyperglycemia and accumulation of large lipid droplets in the fat body [131]. Diabetes in these flies was rescued by PLP administration [130,131]. Interestingly, a wild type human *PDXK* construct introduced in *dPdxk^1^* mutant flies rescued diabetic hallmarks; in contrast, no rescue was observed when human *PDXK* variants (carrying missense mutation impairing catalytic activity) were inserted in *dPdxk^1^* mutant flies. This suggests that the *PDXK* gene could be putatively implied in human diabetes [132] and that PLP-depleted diabetic individuals may be useful for studies aimed at dissecting the molecular mechanisms through which vitamin B6 exerts a protective role against diabetes. 

T2D models obtained by HSD-feeding allowed the discovery of new genes and pathways involved in insulin resistance. For example, HSD-fed flies contributed to confirmation of the involvement of the JAK/STAT pathway in the development of obesity and diabetes [133]. It was recently found that the loss of the JAK/STAT pathway receptor domeless in the fat body was able to reverse, at least in part, the dysmetabolism induced by a high sugar diet [134]. 

Moreover, HSD-fed animals helped to further confirm the involvement of stress Jun N-terminal kinase (JNK) signaling in insulin resistance [135]. In particular, it was observed that a target of JNK signaling, the lipocalin Neural Lazarillo (NLaz)—the ortholog of human apolipoprotein D (ApoD)—is strongly expressed in HSD flies; in contrast, animals heterozygous for an NLaz null mutation are fully protected from HSD-induced insulin resistance [98]. Notably, the overexpression of the mammalian ortholog of NLaz leads to glucose intolerance, insulin resistance and hepatic steatosis in mice [136], thus suggesting that flies represent a suitable model to identify additional factors involved in insulin resistance and potentially targetable by drugs.

Another pathway implicated in diabetes concerns the tryptophan metabolism. It was proposed that the impairment of this metabolic pathway may contribute to insulin resistance, leading to the accumulation of diabetogenic compounds which interfere with insulin action [137,138]. Studies in *Drosophila* supported this hypothesis. It was indeed shown that HSD-fed mutants of the TRP 2,3-dioxygenase (TDO) enzyme (*vermilion* gene)—required for the conversion of tryptophan in kynurenine—displayed reduced insulin resistance, thus reinforcing the hypothesis that associates tryptophan metabolism with diabetes [139].

### 3.4. Monogenic Diabetes Fly Models

MODY has also been modeled in *Drosophila*. In particular, MODY1 flies were generated by inducing the loss of the HNF4 factor, a close ortholog of human HNF4A. MODY1 flies exhibited hyperglycemia, sugar intolerance and decreased production of DILPs [140]. The MODY1 fly model allowed dissection of the HNF4 functions, unveiling that this protein contributes to glucose homeostasis maintenance by regulating the expression of the *GCK* fly ortholog (*Hex-C*) which is required for DILP secretion. In addition, HNF4 impacts on glucose homeostasis also as downstream target of Sir2 deacetylase [141], previously identified as a regulator of lipid storage in *Drosophila* [142]. HNF4 also regulates the expression of oxidative phosphorylation genes required for the larval-to-adult transition [140] and mediates the rapid conversion of persisting larval fat stores into hydrocarbons, shortly after the eclosion [143].

A model of MODY2 was generated by Mascolo and co-workers [144]. Differently from mammals which possess only one *GCK* gene and different tissue-specific isoforms, *Drosophila* has two main *GCK* orthologs: *Hex-C* expressed in the fat body and *Hex-A* expressed in IPCs, which perform the functions of hepatic and pancreatic mammalian GCK, respectively [144]. Moreover, *Hex-A* is required for insulin secretion and to trigger the expression of *Hex-C* in the fat body [144]. RNAi-induced silencing of *Hex-A* or *Hex-C* genes resulted in diabetic phenotypes similar to those observed in MODY2 patients. Interestingly, this *Drosophila* MODY2 model has been useful to discover that hyperglycemia due to GCK loss causes chromosome aberrations through the formation of advanced glycation end-products (AGEs) and reactive oxygen species (ROS). This finding indicates that, although MODY2 rarely produces complications, it may impact on genome integrity [144].

## 4. *Drosophila* as a Mean to Validate Human Candidate Genes

*Drosophila* has been employed also to validate human candidate genes identified through GWAS screenings (Table 2). Pendse and collaborators were the first to perform a screening by examining 83 orthologs of 71 candidate human genes, testing sucrose-dependent toxicity in RNAi flies. From this analysis, it emerged that a large number of fly orthologs play an important role in *Drosophila* tolerance to high dietary sucrose, thus reinforcing their implication in diabetes. In particular, *Drosophila* ortholog of the *haematopoietically expressed homeobox* (*HHEX*) gene, encoding a transcription factor, was reported to be expressed in the fat body and its loss resulted in insulin resistance, hyperglycemia and systemic decreased levels of triglycerides. This result led to hypothesize that *dHHEX* may play a role in determining the capacity of the fly to store energy as triglycerides [145]. 

Another screening was performed by Peiris and collaborators, with the aim to identify *Drosophila* orthologs of T2D-risk genes, specifically involved in insulin secretion. By examining 14 candidates, authors identified three genes, *BCL11A*, *SIX3* and *PRC1*, as regulators of human beta cell function. A further characterization of *BCL11A* revealed that its loss in primary human islet cells leads to enhanced insulin secretion. Accordingly, gene expression profiling revealed that *BCL11A* regulates multiple genes involved in insulin exocytosis [146].

## 5. Screening to Isolate New Genes Involved in Diabetes

Other screenings were performed to isolate new functions associated with diabetes (Table 2). Ugrankar and co-workers examined about 1000 genes involved in glucose metabolism—inactivated by RNA interference in the fat body or muscles—and found about 160 candidate genes linked to hyperglycemia. In particular, authors focused on *CSNK1α1*, a gene encoding the alpha subunit of casein kinase 1 and validated its implication in glucose metabolism, showing that heterozygous and homozygous mutants for the murine ortholog developed diabetes [99].

To dissect the pathways underlying the process of insulin secretion, Cao and colleagues used laser microdissection and mRNA sequencing, and characterized the transcriptome of larval IPCs. This work unveiled that *Unc-104/Kif1a*, a kinesin-3 microtubule motor, previously known to transport synaptic vesicles [147,148,149], is involved in the transport of insulin-containing vesicles along the axons of IPCs [150]. In addition, starting from the notion that Rab proteins, members of the family of Ras-like GTPases, control many cellular trafficking paths [151], the authors also analyzed 31 dominant-negative Rab proteins, for their effects on insulin production or secretion, finding that Rab1 is crucial for DILP trafficking in IPCs [150].

Zhang and collaborators performed a genetic screen to identify regulators of insulin sensitivity. Overexpression of FOXO, a gene working in the insulin pathway (Figure 4), responds to changes in insulin-like signaling in a highly sensitive manner and produces a phenotype consisting of small rough eyes [152]. Thus, during a screening for FOXO overexpression modifiers, the authors discovered a cross-talk between the epidermal growth factor receptor (EGFR)–activated MAPK/ERK and insulin signaling pathways, suggesting that such a regulatory mechanism—which involves transcriptional control of insulin-like receptor gene mediated by ETS-1 transcription factor *Pointed*—is utilized in vivo to maintain circulating glucose at appropriate levels [153]. 

A GWAS screening was done by He and collaborators to isolate genes involved in the beta cell destruction triggered by unfolded mutant insulin [154]. The expression of the mutant human pre-pro-insulin (hINS(C96Y)) in the eye imaginal disc disrupts eye development and results in a reduced adult eye, mimicking beta cell death [155]. He and colleagues used this model to test inbred lines derived from a natural population (*Drosophila melanogaster* Genetic Reference Panel, DGRP [156]) and screen for enhancers of this phenotype, finding a continuous, highly heritable distribution of eye-degeneration phenotypes. Then, they performed a GWAS screening searching for SNP variants in these DGRP lines and identified the *sulfateless* (*sfl*) gene—involved in the heparan sulfate biosynthetic pathway—as the strongest association with the eye reduction phenotype. *sfl* RNAi lines, as well as RNAi lines for other genes in the same pathway, such as *tout-velo* (*ttv*) and *brother of tout-velo* (*botv*), confirmed this result, implicating HS-modified proteins in the response to protein misfolding. Interestingly, this study revealed that although the model of NDM in the fly is monogenic, the severity of the disease trait is sensitive to genetic background, like a complex trait [154].

## 6. How to Study Diabetes Complications in *Drosophila*

Hyperglycemia is thought to increase the production of ROS, altering a series of downstream pathways such as polyol pathway flux, advanced glycation end-product formation, protein kinase C activation and hexosamine pathway flux [157]. *Drosophila* has been used for exploring specific aspects involved in diabetes complications such as heart defects, retinal damage and diabetic nephropathy (Table 2). Na and colleagues generated a model of HSD-fed flies to study diabetes-induced hearth dysfunctions [158]. They first validated the model by showing that the insulin and P38 MAPK pathways, which mediate heart dysfunction in mammals [159,160], modulate HSD-induced heart defects in *Drosophila* as well. Then, they showed that reducing the activity of either O-linked beta-N-acetylglucosamine transferase (OGT) or glutamine-fructose-6-phosphate transaminase (GFAT) rescued heart failure caused by HSD [158]. This finding indicates that the hexosamine biosynthetic pathway is important for mediating progressive heart defects, revealing potential targets for future therapies.

Interestingly, a *Drosophila* model to study glucose-induced retinal neurodegeneration was generated by Catalani and co-workers. They found that hyperglycemia induced by HSD led to eye defects, apoptosis/autophagy dysregulation, oxidative stress and visual dysfunctions [161]. This model offers the opportunity to study the molecular mechanisms and the pathophysiology of neuroretinal alterations that characterize diabetic patients at the early stages of the disease. 

Diabetic nephropathy (DN) is a major secondary complication that leads to glomerular and renal tubular dysfunction. A recent study indicated that HSD-fed *Drosophila* may be used as a model for identifying genes and mechanisms of renal tubular dysfunction in DN [162]. The authors found that fly Malpighian tubules, a functional equivalent of the vertebrate kidney, recapitulate many endpoints of diabetes-mediated renal tubular dysfunction, including AGEs-receptor for AGEs (RAGE) signaling, apoptosis and expression of genes involved in pathways associated to DN. This finding established a suitable model for dissecting the mechanisms at the basis of this pathology.

**Table 2:** Schematization of the different exploited approaches to study diabetes using *Drosophila* as an experimental model.

***Drosophila* Diabetes Models**		
**Type**	**Generation Method**	**Ref**
T1D	IPC ablation	[112,113,114]
T1D	*Dilp 1-5* gene deletion	[116]
T1D	Dominant negative Hsc70-3 expression	[121]
T2D	HSD feeding	[98,122]
T2D	IIS gene silencing	[105,124,125,126]
T2D	*Pdxk* mutations	[130]
T2D	*Sgll* gene silencing	[131]
MODY1	*dHNF4* gene silencing	[140]
MODY2	*HexA* or *HexC* gene silencing	[144]
**Diabetes Candidate Genes Validated in *Drosophila***		
**Candidate Gene**	**Diabetes Type**	**Ref**
*HHEX*	T2D	[145]
*BCL11A*	T2D	[146]
**Screenings Performed in *Drosophila* to Isolate New Diabetes-Related Genes**		
**Gene**	**Pathway**	**Ref**
*CSNK1α1*	Glucose metabolism	[99]
*Unc-104*	Insulin trafficking	[150]
*Rab1*	Insulin trafficking	
*Pointed*	Glucose level maintenance	[153]
*sulfateless*	Beta cell destruction	[154]
**Diabetes Complications *Drosophila* Models**		
**Complication**	**Diabetes Type**	**Ref**
Heart dysfunction	HSD-induced T2D	[158]
Retinal degeneration	HSD-induced T2D	[161]
Diabetic nephropathy	HSD-induced T2D	[162]

## 7. Diabetes and Cancer Risk in *Drosophila*

Growing evidence indicates that diabetic patients present an increased risk to develop cancer, although molecular mechanisms behind this correlation are mostly unknown [163,164]. Therefore, it is crucial try to identify genes and pathways whose impairment could be at the base of the individual’s cancer susceptibility. 

A causative link between diabetes and cancer has also been observed in *Drosophila* by Hirabayashi and colleagues. The authors provided evidence that HSD can increase the frequency of primary and secondary tumors in a *Drosophila* cancer model generated by the simultaneous overexpression of Ras and Src oncoproteins [165]. In addition, they showed that whereas most of the tissues displayed insulin resistance, Ras/Src tumors retained insulin pathway sensitivity, displayed an enhanced glucose uptake and counteracted apoptosis. Consistently, the authors proposed a model in which HSD induces increased Wingless/Wnt pathway activity, which in turn upregulates the expression of the insulin receptor gene and consequent insulin sensitivity [165]. These results indicated that *Drosophila* represents a suitable model for future studies aimed at gaining insight on the relationship between diabetes and cancer.

It is known that diabetes can establish, over time, a status of oxidative stress, consisting of raised levels of ROS, decreased levels of natural antioxidants and reduced DNA repair efficiency [166,167,168]. In line with this notion, diabetes was associated to chromosome breakage and telomere shortening, raising the hypothesis that DNA damage could be one of the mechanisms connecting diabetes to cancer [169]. 

We demonstrated that the loss of *Drosophila* proteins working in the insulin pathway, such as Inr, Chico and Akt, produced glucose-sensitive chromosome aberrations [125]. More interestingly, the treatment of these flies with an inhibitor of vitamin B6 strongly enhanced DNA damage [125]. Hence, transferring to humans, these data not only give robust support to the hypothesis that hypoglycemia strongly impairs genome integrity, but also strengthen the notion that a reduced availability of B6 vitamin may behave as a cancer risk factor for diabetic patients. These data are consistent with the growing evidence that micronutrient levels could strongly impact on genome integrity, leading to cancer development [170,171]. Taken together, these observations strongly suggest that *Drosophila* is a very suitable tool to screen and identify diet components able to mitigate cancer risk in diabetic patients. 

## 8. Conclusions

**Figure 5:**
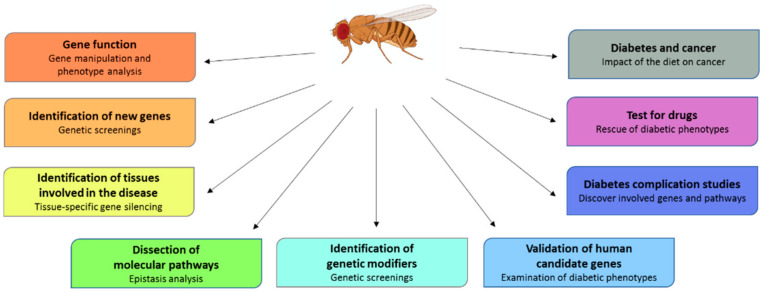
Advantages and applications of *Drosophila* as a diabetes model. *Drosophila* offers the possibility of studying diabetes by employing numerous approaches to increase DM knowledge and provide translational benefits.

Unveiling the genetic basis of diabetes is a challenge associated to great goals such as the predictability of the risk, the development of diagnostic biomarkers and the application of personalized cures. The rapid development of avant-garde experimental strategies and tools has accelerated the progression of research, allowing the association of several genes to diabetes. However, unfortunately, the studies carried out to date did not give expected results for the prediction of the risk of multigenic diabetes. What emerged is that it is essential to exploit several combined approaches in order to obtain better and faster results. Once the association of a gene with the disease has been established, major efforts are needed to address the comprehension of the roles of candidate genes and their relative pathways. This knowledge will be the basis for the development of more focused therapies in the near future. We expect that the use of *Drosophila*, an invaluable model organism to perform genetic screenings, monitor disease progression and test disease modifiers (Figure 5), will be helpful to perform functional studies and to dissect new identified pathways, contributing to reinforce the idea that personalized care does not remain a dream.

## Figures and Tables

**Table 1:** MODY subtypes and their related genes.

MODY Subtype	Gene
1	*HNF4A*
2	*GCK*
3	*HNF1A*
4	*PDX1*
5	*HNF1B*
6	*NEUROD1*
7	*KLF11*
8	*CEL*
9	*PAX4*
10	*INS*
11	*BLK*
12	*KCNJ11*
13	*ABCC8*
14	*APPL1*

## Data Availability

Not applicable.
